# Comment on molybdenum polyoxo clusters: from the ‘Blues’ to the ‘Reds’

**DOI:** 10.1107/S2053229622004491

**Published:** 2022-05-09

**Authors:** Bernt Krebs

**Affiliations:** a Westfälische Wilhelms-Universität Münster, Institut für Anorganische und Analytische Chemie, Corrensstrasse 28/30, D-48149 Münster, Germany

**Keywords:** giant assemblies, building blocks, lanthanides, molecular wheels, molecular cages, molecular hybrids, polyoxomolybdate

## Abstract

Molybdenum ‘Blues’, ‘Browns’ and the recently discovered ‘Reds’ are unquestionably some of the most impressive nanoscale architectures in POM chemistry. In this context, Al-Sayed *et al.* (2022) recently made a significant contribution, reporting a new binding mode of an organic hybridization reagent in mixed-valence polyoxomolybdates.

Molybdenum ‘Blues’, ‘Browns’ and the recently discovered ‘Reds’ are unquestionably some of the most impressive nanoscale architectures in polyoxometalate (POM) chemistry (Müller & Gouzerh, 2012 ▸; Lin *et al.*, 2020 ▸). These three polyoxomolybdate classes, with the general formula [*X_a_Y_b_* H_*c*_Mo^VI^_*x*_Mo^V^_*y*_O_*z*_(H_2_O)_*v*_]^*n*−^ (*a* and *b* = number of organic ligands and heteroelements, respectively; *c* = degree of protonation; *x* and *y* = number of Mo^VI^ and reduced Mo^V^ centres, respectively) are classified as gigantic mixed-valence (Mo^V/VI^) polyoxomolybdate clusters with various topologies ranging from ‘wheels’ to ‘balls’, ‘cages’ and the ‘blue lemon’, known to be the largest inorganic mol­ecule to date (Lin *et al.*, 2020 ▸; Müller & Gouzerh, 2012 ▸). While the subclasses of molybdenum ‘Blues’, ‘Browns’ and ‘Reds’ have the reduction of an acidified solution containing orthomolybdate ([MoO_4_]^2−^) or hepta­molybdate (H_*x*_[Mo_7_O_24_]^(6–*x*)–^) in common, they can be distinguished by three characteristic features. Firstly, ‘Blues’, ‘Browns’ and ‘Reds’ exhibit distinct degrees of reduction, with the ‘Blues’ being the least (∼18%) and the ‘Reds’ the most (up to ∼81%) reduced representatives (Ribó *et al.*, 2022 ▸).

Secondly, in terms of molybdenum building blocks, wheel-shaped molybdenum ‘Blues’ are composed of {MoO_6_} ({Mo_1_}), corner-sharing {Mo_2_O_11_} ({Mo_2_}) and the essential building block {Mo_8_O_35_} ({Mo_8_}), while the ball-shaped ‘Browns’ consist of {Mo_1_}, edge-sharing {Mo_2_O_10_} ({Mo_2_′}) and {Mo_6_O_27_} ({Mo_6_}) units (Fig. 1 ▸) (Müller & Gouzerh, 2012 ▸).

Remarkably, molybdenum ‘Reds’ exclusively comprise {Mo_1_} and {Mo_2_′} units (Fig. 1 ▸) (Lin *et al.*, 2020 ▸), hence requiring a comparably trickier synthetic approach than their blue counterparts, which necessitates the use of additional cluster-stabilizing coordinated ions (*e.g.* SO_3_^2−^, 3*d*- and 4*f*-metal ions) (Lin *et al.*, 2020 ▸; Ribó *et al.*, 2022 ▸) to aggregate the {Mo_1_} and dumbbell-shaped {Mo_2_′} into larger clusters, consequently promoting mol­ecular growth (Ribó *et al.*, 2022 ▸).

Contrary to molybdenum ‘Reds’, additional ions (*e.g.* 3*d*- and 4*f*-metal ions) react differently in ‘Blue’ systems as they are utilized here for fine-tuning the mol­ecular shape (Garrido Ribó *et al.*, 2020 ▸) and alternating the mol­ecule’s physical characteristics rather than spanning the cluster framework.

For instance, large electrophilic ‘open-shell’ metal centres, such as 4*f*-metal ions, alter the overall charge, mol­ecular shape and size, and always cause a symmetry reduction of molybdenum ‘Blues’ when introduced into their frameworks (Al-Sayed & Rompel, 2022 ▸). In ‘Blue’ systems, however, 3*d*-metal ions seem to behave in a significantly opposite manner from their 4*f* counterparts considering their relatively small size which leaves them without any impact on the assembly of the {Mo_1_}, {Mo_2_} and {Mo_8_} building blocks (Fig. 1 ▸).

Thirdly, in terms of functionalizability, the ‘Blues’ and ‘Browns’ can be organically functionalized, resulting in reaction vessels for studying confined mol­ecules. Organic hybridization is typically accomplished by attaching carboxyl groups to the cluster and nitro­gen-containing pendant groups in carb­oxy­lic acids. Such constructs are capable of promoting mol­ecular growth and triggering the formation of various guest@host architectures (Xuan *et al.*, 2017 ▸, 2019 ▸; Imai *et al.*, 2009 ▸). In stark contrast to their ‘Blue’ counterparts, nothing is yet known about the organic hybridizability of molybdenum ‘Reds’. In the event of a molybdenum ‘Red’ hybridization, the dumbbell-shaped and edge-sharing {Mo_2_′} (Fig. 1 ▸) building blocks would have to be functionalized organically.

In this context, Al-Sayed *et al.* (2022 ▸) recently made a significant contribution, reporting a new binding mode of an organic hybridization reagent in mixed valence polyoxomolybdates (Al-Sayed *et al.*, 2022 ▸). The organic functionalization of a dumb­bell-shaped and edge-sharing {Mo_2_′} building block occurred through chelation of a pyridine derivative, as shown by the isolation of the [Mo_2_O_2_(OH)_4_(C_6_H_4_NO_2_)_2_]^2+^ unit (Fig. 2 ▸) acting as a charge-balancing dication for the molybdenum ‘Blue’ cluster Na_4_[Mo_2_O_2_(OH)_4_(C_6_H_4_NO_2_)_2_]_2_[Mo_120_Ce_6_O_366_H_12_(OH)_2_(H_2_O)_76_]∼200H_2_O. As ‘Reds’ comprise a multitude of {Mo_2_′} units, the outstanding work of Al-Sayed *et al.* (2022 ▸) represents a major synthetic step forward, since it showcases the first example of the *N*,*O*-chelation of {Mo_2_′} units (Fig. 2 ▸).

To understand the fundamental principles of a multicomponent system driving the assembly of these aesthetically pleasing constructions, as well as the prospects for forming novel ones, is a major undertaking. Identifying novel steps in the formation process of mixed-valence polyoxomolybdates, such as an effect of a structure-directing ligand or implementing a unique functionalization of building blocks, will enable the construction of POM clusters with unprecedented sizes and topologies, thereby shedding light upon the mostly elusive self-assembly mechanisms, ultimately perhaps paving the way towards novel POM architectures with nuclearities surpassing the famous Na_48_[H_*x*_Mo_368_O_1032_(H_2_O)_240_(SO_4_)_48_]∼1000H_2_O (Mo_368_) ‘blue lemon’ (Müller *et al.*, 2002 ▸).

More than two decades separate the discovery of the ‘Blues’ and the ‘Reds’. Is that the end of the molybdenum classes, or will the synthetic chemists have yet another new colour to enjoy?

## Figures and Tables

**Figure 1:**
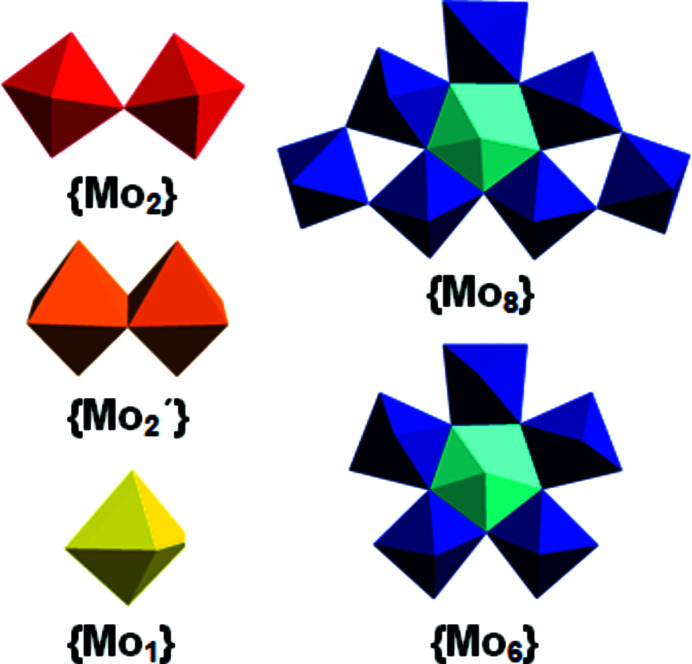
Polyhedral representation of the building blocks in mixed-valence polyoxomolybdates. Colour code: {MoO_6_} ({Mo_1_}), yellow; corner-sharing {Mo_2_O_11_} ({Mo_2_}), red; edge-sharing {Mo_2_O_10_} ({Mo_2_′}, ochre; {Mo_6_O_27_} ({Mo_6_}) and {Mo_8_O_35_} ({Mo_8_}), blue, with the central {MoO_7_} unit in cyan.

**Figure 2:**
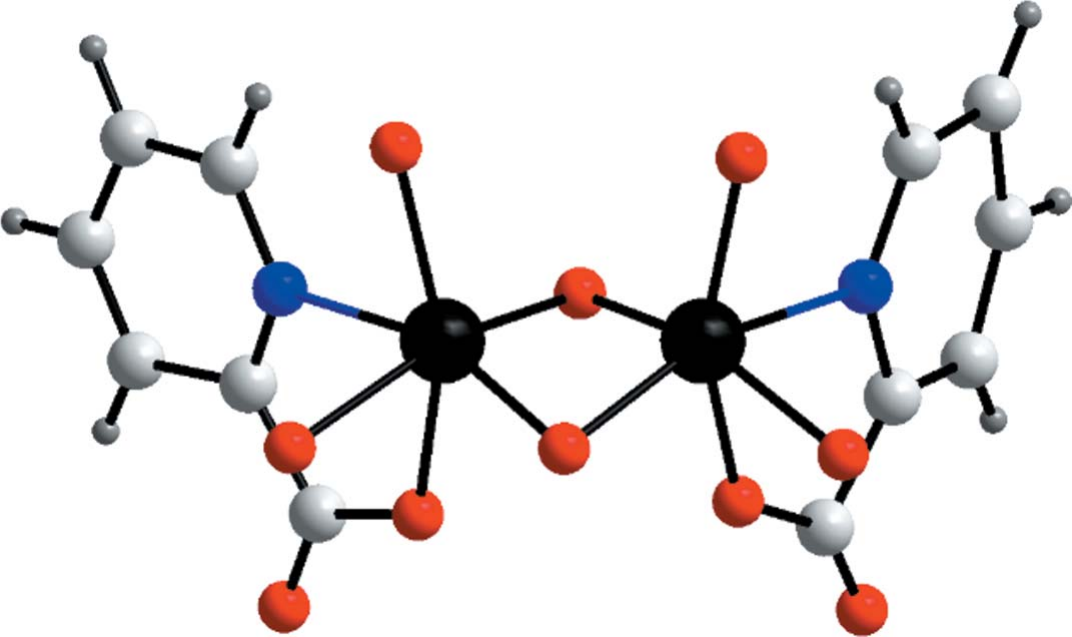
Ball-and-stick representation of the organofunctionalized [Mo_2_O_2_-(OH)_4_(C_6_H_4_NO_2_)_2_]^2+^ unit acting as a charge-balancing dication for the Japanese rice-ball-shaped Molybdenum Blue Na_4_[Mo_2_O_2_(OH)_4_(C_6_H_4_NO_2_)_2_]_2_[Mo_120_Ce_6_O_366_H_12_(OH)_2_(H_2_O)_76_]∼200H_2_O. Colour code: Mo black, O red, C grey, N blue and H white.
